# Associations of Proteinuria Trajectories with Kidney Failure and Death in Individuals with CKD

**DOI:** 10.34067/KID.0000000849

**Published:** 2025-06-26

**Authors:** Avi G. Aronov, Ashish Verma, Ana C. Ricardo, Tanika N. Kelly, Sushrut S. Waikar, James P. Lash, Anand Srivastava, the CRIC Study Investigators, Amanda H. Anderson

**Affiliations:** 1 Department of Medicine, University of Illinois Chicago, Chicago, Illinois; 2 Section of Nephrology, Department of Medicine, Boston Medical Center, Boston University Chobanian and Avedisian School of Medicine, Boston, Massachusetts; 3 Division of Nephrology, Department of Medicine, University of Illinois Chicago, Chicago, Illinois

**Keywords:** CKD, ESKD, kidney failure, proteinuria, risk factors

## Abstract

**Key Points:**

- Clinicians may repeat proteinuria measurements multiple times but may overlook the significance of temporal patterns of proteinuria.
- Trajectory analyses identified four discrete proteinuria subgroups: low-slowly rising, high-slowly rising, regressing, and rapidly rising.
- Trajectories of proteinuria identify subgroups of patients with CKD who have increased risks of ESKD and death independent of known risk factors.

**Background:**

Despite repeating proteinuria measurements multiple times during the clinical course of a patient with CKD, clinicians may overlook the significance of temporal patterns of proteinuria. In addition, it is unclear whether proteinuria trajectories identify subpopulations with varying risks of adverse clinical outcomes.

**Methods:**

We used group-based trajectory modeling to identify proteinuria trajectories on the basis of annual urine protein–creatinine ratio (UPCR) measurements in 3209 participants of the Chronic Renal Insufficiency Cohort study who were alive and did not reach ESKD within 3 years of study entry. Multivariable-adjusted Cox proportional hazards models tested the associations of UPCR trajectories with ESKD and death in those who survived beyond the third annual visit.

**Results:**

Trajectory analyses identified four discrete groups on the basis of annual UPCR measurements: low-slowly rising (*n*=1528), high-slowly rising (*n*=1363), regressing (*n*=114), and rapidly rising (*n*=204). Compared with the low-slowly rising proteinuria trajectory group, participants in the other proteinuria trajectory groups had lower socioeconomic status, a greater prevalence of comorbid conditions, and lower eGFR. During a median follow-up of 8.6 years, 547 participants progressed to ESKD, and 836 participants died. Compared with the low-slowly rising group, all proteinuria trajectory groups were associated with higher risks of subsequent ESKD, but only the high-slowly rising group was associated with a higher risk of death.

**Conclusions:**

Trajectories of repeated proteinuria measurements identify subgroups of patients with CKD who have increased risks of ESKD and death independent of known risk factors.

## Visual Abstract

**Figure:**
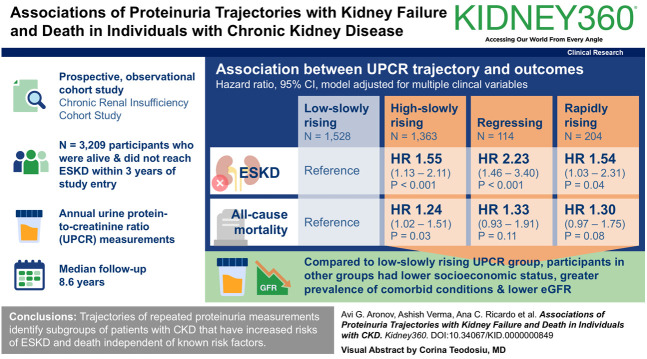

## Introduction

Despite the high filtered load, the kidneys only allow a small amount of protein in urine.^1^ Owing to the efficiency of the kidney filtration system, abnormal protein levels in urine are a critical biomarker to diagnose and assess the severity of CKD.^2,3^ Quantifying proteinuria guides clinical decision making, and its incorporation into risk prediction models enables the ability to estimate the risk of progression to ESKD.^4,5^ While proteinuria remains a strong independent risk factor of adverse kidney outcomes, clinicians rely on a single-time point measurement to assess risk despite repeating the measurement multiple times in the clinical course of an individual patient. Reliance on a single-time point measurement may overlook temporal patterns of proteinuria. In addition, it is unclear whether trajectories of proteinuria identify subpopulations with varying risks of adverse clinical outcomes to target for therapeutic intervention.

Recent insights suggest that changes in proteinuria over time, rather than isolated measurements, may provide significant prognostic value for adverse clinical outcomes.^6–9^ Analysis of proteinuria trajectories may detect heterogeneity in a population and identify subgroups of patients whose evolution of proteinuria is significantly different than the population mean and confers varying risks of adverse clinical outcomes. Building on these insights, we performed a prospective cohort study among participants with mild-to-severe CKD in the Chronic Renal Insufficiency Cohort (CRIC) Study to test whether proteinuria trajectories identify discrete subgroups of patients with CKD and whether these discrete subgroups have varying risks of ESKD and death.

## Methods

### Source Population

The CRIC Study is a prospective, observational cohort study of patients with mild-to-severe CKD designed to examine risk factors of progression of CKD, development of cardiovascular disease (CVD), and mortality. During phase 1, the CRIC Study enrolled 3939 men and women aged 21–74 years between 2003 and 2008 across seven clinical centers in the United States. An additional 1560 participants were recruited between 2013 and 2015 during phase 2. Patients were included if they met specific age-defined criteria for an eGFR of 20–70 ml/min per 1.73 m^2^. Black and Hispanic participants were oversampled.^10,11^ Exclusion criteria included a history of dialysis exceeding 1 month, kidney transplantation, New York Heart Association class 3 or 4 congestive heart failure, active treatment with immunosuppressants for GN, or a diagnosis of cirrhosis or polycystic kidney disease. The study protocol was approved by the Institutional Review Boards of all participating centers and is in accordance with the principles of the Declaration of Helsinki. All CRIC Study participants provided written informed consent.

### Study Population

**Figure 1:**
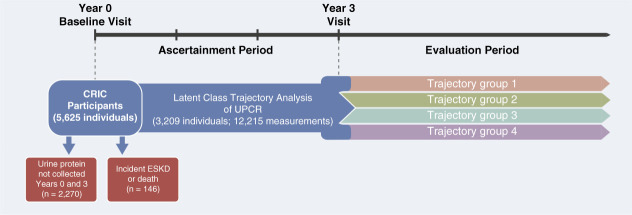
**Description of study design.** The proteinuria trajectories were derived during the ascertainment period (baseline visit to year 3). Among 5625 participants, 2270 participants were excluded for not having UPCR collected at year 0 and year 3. Participants who progressed to ESKD or died in the ascertainment period were also excluded (*n*=146), which resulted in 3209 eligible participants (12,215 UPCR measurements) to derive proteinuria trajectory subgroups. The evaluation period included follow-up of participants that began after the year 3 visit. CRIC, Chronic Renal Insufficiency Cohort; UPCR, urine protein–creatinine ratio.

To identify subgroups of patients with CKD who had varying patterns of proteinuria over a 3-year ascertainment period and to determine the associations of the subgroups with adverse clinical outcomes, we included 3209 CRIC Study participants who survived beyond their fourth annual study visit (baseline through year 3 visit) without progressing to ESKD (Figure 1). All included study participants also had urine protein–creatinine ratio (UPCR) levels at the baseline and year 3 visit.

### Exposures

The primary exposures were grouped trajectories of proteinuria, which we formed from repeated UPCR measurements. Urine samples were collected annually from the baseline visit through year 3, which yielded up to four UPCR values per participant. The mean±SD number of UPCR values was 3.8±0.4 per participant. Spot urine protein and creatinine values were obtained from 24-hour or random spot urine collections. If both were available, the 24-hour UPCR was used. Total urine protein was determined using the turbidimetric method with benzethonium chloride, and urine creatinine was determined using the kinetic rate Jaffe method. The intra-assay coefficients of variation were 3.8% and 2.1% for total urine protein and urine creatinine, respectively. UPCR (g/g creatinine) was calculated as spot urine protein concentration divided by spot urine creatinine concentration.^12^

### Outcomes

The primary outcomes were ESKD, defined as the initiation of dialysis or kidney transplantation, and all-cause mortality. ESKD status was confirmed by cross-linking participants with the United States Renal Data System.^13^ Mortality data were verified through the examination of death certificates. Study participants were followed longitudinally, from their enrollment date until an event of interest, voluntary withdrawal from the study, loss to follow-up, or the end of the study period in December 2021.

### Ascertainment of Covariates

We assessed covariate data encompassing sociodemographic characteristics, medical history, lifestyle behaviors, medications, standardized BP measurements, anthropometric data, and laboratory results. We used the race-free CKD Epidemiology Collaboration 2021 equation to calculate eGFR.^14^

### Statistical Analyses

We used group-based trajectory modeling to categorize participants who were alive and did not reach ESKD within 3 years of study entry on the basis of annual measurements of UPCR.^15^ We used the “hlme” function from the “lcmm” package in R, which accommodates discrete mixture modeling to delineate clusters of longitudinal data series representing distinct trajectories of UPCR levels.^16^ This method relies on a semiparametric group-based modeling strategy, which incorporates hierarchical and latent growth curve modeling, and it assumes that there are multiple trajectory groups within a population. We applied a cubic polynomial function with random intercepts that allowed for a nonproportional random-effects structure to accurately capture the dynamic nature of UPCR changes over time. We evaluated models with different numbers of trajectory groups for model fit, which we assessed with an average posterior probability of assignment, odds of correct classification, relative entropy, and Bayesian information criterion (Supplemental Table 1).^17–19^ On the basis of the model fit criteria and visual appearance of the trajectories, we identified four proteinuria trajectory groups. We assigned participants to the trajectory group for which they had the highest posterior predicted probability.^15^ The mean posterior probabilities were 85.8%, 87.4%, 81.9%, and 78.0% for the low-slowly rising, high-slowly rising, regressing, and rapidly rising proteinuria trajectory groups, respectively (Supplemental Figure 1).

After derivation of the proteinuria trajectory groups within 3 years of study entry, we summarized descriptive statistics at the year 3 visit according to trajectory group membership as mean±SD or median (interquartile range) for continuous variables and as percentages for frequency distribution for categorical variables. We used chi-square tests to compare frequency distributions of categorical variables by proteinuria trajectory groups. We used analysis of variance and Kruskal-Wallis tests to evaluate normal and non-normally distributed continuous variables with proteinuria trajectory groups.

We used Cox proportional hazards regression to test the associations between proteinuria trajectory groups and risks of ESKD and all-cause mortality after the 3-year ascertainment period. We set the survival time (time 0) to begin with the participant's fourth annual visit (year 3 visit). All covariates were ascertained at the time survival follow-up began. We fit a series of hierarchically adjusted models on the basis of the biological and clinical plausibility of covariates as potential confounders: Model 1 was stratified by clinical site and adjusted for age, sex, race and ethnicity, household income, education, systolic BP, diabetes mellitus, history of CVD, body mass index, current smoking, hemoglobin, and medications (angiotensin-converting enzyme inhibitors [ACEi] or angiotensin II receptor blockers [ARBs], *β*-blockers, lipid-lowering agents, and antiplatelet agents); model 2 included covariates from model 1 and was further adjusted for eGFR at year 3; model 3 included covariates from model 2 and was further adjusted for natural log-transformed UPCR at year 3. We used the “mice” package for multiple imputations using predictive mean matching across ten datasets and 100 iterations to account for missing covariate data.^20^ Additional information about our multiple imputation approach are provided in the Supplemental Methods. All covariates had <5% missing data (Supplemental Table 2). We combined the results across the imputed datasets using Rubin's rules.^21^ We confirmed no violations of the proportional hazards assumption using Schoenfeld residuals. All analyses were completed with R version 4.2.1.

### Sensitivity Analyses

To account for a participant's risk of ESKD or all-cause mortality upon entering the CRIC Study, we adjusted for baseline (year 0) eGFR and UPCR. Because a participant's level of UPCR may differ on the basis of the use of ACEi or ARB, we further adjusted for ACEi or ARB use at the baseline (year 0) visit. In another sensitivity analysis, we increased the ascertainment period to year 4 to evaluate its potential influence on proteinuria trajectory groups and risk estimates. We evaluated whether the competing risk of death changes the association of the proteinuria trajectory groups with subsequent ESKD. Because we used multiple imputation in the primary analyses, we performed a complete case analysis as an additional sensitivity analysis.

## Results

### Proteinuria Trajectory Groups

Table 1 presents year 3 characteristics of the study population. The mean age was 63±10 years, 56% were male, 46% were of White race, 51% had diabetes, mean eGFR was 46±18 ml/min per 1.73 m^2^, and median UPCR was 0.16 (0.06–0.60) g/g creatinine.

**Table 1:** Characteristics of urine protein–creatinine ratio trajectory subphenotypes at study year 3

Characteristics	Overall (*N*=3209)	Proteinuria Trajectory Groups				*P* Value
		Low-Slowly Rising (*n*=1528)	High-Slowly Rising (*n*=1363)	Regressing (*n*=114)	Rapidly Rising (*n*=204)	
Age, yr	63±10	64±9	62±11	61±10	64±9	<0.001
Male	1804 (56%)	761 (50%)	863 (63%)	68 (60%)	112 (55%)	<0.001
**Race and ethnicity**						<0.001
Non-Hispanic Black	1323 (41%)	566 (37%)	613 (45%)	49 (43%)	95 (47%)	
Non-Hispanic White	1492 (46%)	827 (54%)	538 (39%)	48 (42%)	79 (39%)	
Other	394 (12%)	135 (8.8%)	212 (16%)	17 (15%)	30 (15%)	
**Household income, dollars**						<0.001
<20,000	845 (26%)	323 (21%)	421 (31%)	35 (31%)	66 (32%)	
20,000–50,000	820 (26%)	391 (26%)	341 (25%)	25 (22%)	63 (31%)	
50,001–100,000	649 (20%)	332 (22%)	259 (19%)	22 (19%)	36 (18%)	
>100,000	418 (30%)	255 (17%)	133 (9.8%)	18 (16%)	12 (5.9%)	
Do not wish to answer	477 (15%)	227 (15%)	209 (15%)	14 (12%)	27 (13%)	
**Education level**						<0.001
Less than high school	498 (16%)	171 (11%)	266 (20%)	16 (14%)	45 (22%)	
High school graduate	572 (18%)	240 (16%)	268 (20%)	24 (21%)	40 (20%)	
Some college	956 (30%)	458 (30%)	407 (30%)	34 (30%)	57 (28%)	
College graduate or higher	1181 (37%)	657 (43%)	422 (31%)	40 (35%)	62 (30%)	
Current smoking	347 (11%)	131 (8.6%)	172 (13%)	17 (15%)	27 (13%)	0.001
BMI, kg/m^2^	32±8	32±7	33±8	33±8	33±7	0.016
Systolic BP, mm Hg	126±20	121±18	131±21	121±17	138±23	<0.001
Diabetes mellitus	1651 (51%)	611 (40%)	829 (61%)	67 (59%)	144 (71%)	<0.001
CVD^a^	1215 (38%)	493 (32%)	576 (42%)	46 (40%)	100 (49%)	<0.001
ACEi/ARB (baseline)	2193 (69%)	936 (62%)	1014 (75%)	84 (74%)	159 (78%)	<0.001
ACEi/ARB (year 3)	2195 (69%)	976 (64%)	996 (73%)	88 (78%)	135 (66%)	<0.001
Diuretic	1716 (54%)	742 (49%)	751 (55%)	81 (72%)	142 (70%)	<0.001
Lipid-lowering medication	2199 (69%)	1006 (66%)	957 (71%)	87 (77%)	149 (73%)	0.007
Antiplatelet	1745 (55%)	814 (54%)	748 (55%)	62 (55%)	121 (59%)	0.40
*β*-blocker	1618 (51%)	672 (44%)	757 (56%)	66 (58%)	123 (60%)	<0.001
Hemoglobin, g/L	12.8±1.8	13.2±1.6	12.6±1.9	12.4±1.7	12.3±1.8	<0.001
eGFR, ml/min per 1.73 m^2^ (baseline)	50±15	54±14	48±15	49±15	43±13	<0.001
eGFR, ml/min per 1.73 m^2^ (year 3)	46±18	52±16	41±17	41±18	40±18	<0.001
UPCR, g/g (baseline)	0.12 (0.05–0.43)	0.05 (0.04–0.08)	0.43 (0.19–1.13)	1.08 (0.50–2.28)	0.12 (0.06–0.30)	<0.001
UPCR, g/g (year 3)	0.16 (0.06–0.60)	0.06 (0.05–0.10)	0.56 (0.27–1.38)	0.10 (0.05–0.19)	1.43 (0.55–3.28)	<0.001

All variables ascertained at the year 3 visit unless noted otherwise. Data are presented as mean±SD, median (interquartile range), or count with frequencies (%). ACEi, angiotensin-converting enzyme inhibitor; ARB, angiotensin II receptor blocker; BMI, body mass index; CVD, cardiovascular disease; UPCR, urine protein–creatinine ratio.

a Defined as congestive heart failure, myocardial infarction, cerebrovascular accident, or peripheral vascular disease.

**Figure 2:**
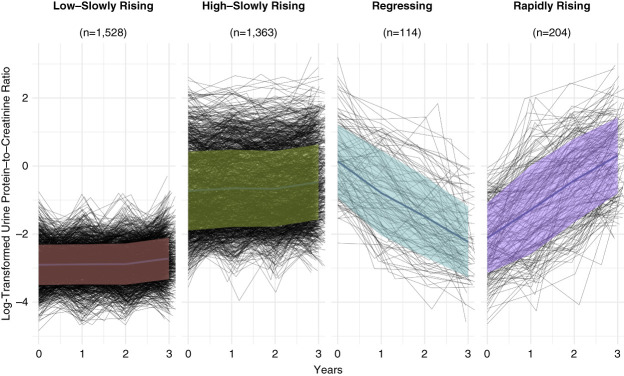
**Trajectory modeling identified four distinct proteinuria trajectory groups.** The analysis included 3209 participants, categorized into low-slowly rising (*n*=1528), high-slowly rising (*n*=1363), regressing (*n*=114), and rapidly rising (*n*=204) proteinuria trajectory groups. The solid lines represent the mean natural log-transformed UPCR within each subphenotype, while the shaded ribbons indicate the variation within 1 SD of the mean.

We identified four proteinuria trajectory groups on the basis of the evolution of UPCR and labeled them on the basis of their patterns: low-slowly rising (*n*=1528), high-slowly rising (*n*=1363), regressing (*n*=114), and rapidly rising (*n*=204; Figure 2). Table 1 presents the characteristics of the four proteinuria trajectory groups at the year 3 study visit. From the baseline (year 0) to year 3 visit, UPCR increased minimally in the low-slowly rising and high-slowly rising proteinuria trajectory groups. By contrast, UPCR started higher and decreased in the regressing trajectory group but started lower and increased in the rapidly rising trajectory group.

The regressing trajectory group participants were the youngest (mean age 61 years). Participants in the low-slowly rising proteinuria trajectory group were more likely to be White and have higher household income and levels of education. The low-slowly rising group also had the lowest body mass index (mean 32 kg/m^2^). The rapidly rising trajectory had the highest percentage of patients with a history of CVD (49%), highest prevalence of diabetes (71%), and highest mean systolic BP (138 mm Hg). In addition, median UPCR (1.43 [0.55–3.28] g/g creatinine) was the highest and mean eGFR (40±18 ml/min per 1.73 m^2^) was the lowest in the rapidly rising proteinuria trajectory group compared with the other proteinuria trajectory groups at the year 3 visit. The regressing proteinuria trajectory group had the highest percentage of participants using ACEi or ARB (78%) and diuretic medications (72%).

### Proteinuria Trajectory Groups and Risks of ESKD

During a median follow-up time of 7.4 years, 547 participants progressed to ESKD. Compared with the low-slowly rising proteinuria trajectory group, all proteinuria trajectory groups were significantly associated with subsequent ESKD (Table 2). These associations were attenuated after adjustment for eGFR and natural log-transformed UPCR. Although the magnitudes of association were attenuated, each proteinuria trajectory was statistically significantly associated with subsequent risk of ESKD compared with the low-slowly rising trajectory group. Of these trajectory groups, the regressing proteinuria trajectory group (hazard ratio [HR], 2.23; 95% confidence interval, 1.46 to 3.4) had the greatest magnitude of association with future ESKD compared with the low-slowly rising proteinuria trajectory group. The results were qualitatively unchanged in a sensitivity analysis that adjusted for eGFR and UPCR at baseline (year 0) and for eGFR and UPCR slopes from baseline (year 0) to year 3 (Supplemental Table 3). In an additional sensitivity analysis that re-created the proteinuria trajectory groups from baseline (year 0) to the year 4 visit, the associations of proteinuria trajectory groups with future risk of ESKD were qualitatively similar (Supplemental Table 4).

**Table 2:** Association of proteinuria trajectory groups with risks of ESKD

Trajectory Groups	No. of Events	Events per 1000 Person-Years	Model 1^a^		Model 2^b^		Model 3^c^	
			Hazard Ratio (95% CI)	*P* Value	Hazard Ratio (95% CI)	*P* Value	Hazard Ratio (95% CI)	*P* Value
Low-slowly rising	80	5.6	Reference	—	Reference	—	Reference	—
High-slowly rising	498	63.4	8.07 (6.30 to 10.3)	<0.001	4.59 (3.56 to 5.90)	<0.001	1.55 (1.13 to 2.11)	<0.001
Regressing	35	47.0	5.35 (3.54 to 8.06)	<0.001	2.67 (1.77 to 4.04)	<0.001	2.23 (1.46 to 3.40)	<0.001
Rapidly rising	89	91.6	9.59 (6.97 to 13.2)	<0.001	6.77 (4.89 to 9.38)	<0.001	1.54 (1.03 to 2.31)	0.04

CI, confidence interval.

a Model 1: stratified by clinical centers and adjusted for age, sex, race and ethnicity, household income, education, systolic BP, diabetes, history of cardiovascular disease, body mass index, current smoking, hemoglobin, angiotensin-converting enzyme inhibitors or angiotensin II receptor blockers, *β*-blockers, diuretics, lipid-lowering agents, and antiplatelet agents.

b Model 2: model 1 and further adjusted for eGFR.

c Model 3: model 2 and further adjusted for natural log-transformed urine protein–creatinine ratio.

### Proteinuria Trajectory Groups and Risks of All-Cause Mortality

During a median follow-up time of 8.6 years, 836 participants died. Table 3 presents the multivariable-adjusted associations between proteinuria trajectory groups and all-cause mortality. All proteinuria trajectory groups had statistically significant associations with future all-cause mortality compared with the low-slowly rising proteinuria trajectory group. After adjustment for eGFR and natural log-transformed UPCR, the regressing and rapidly rising proteinuria trajectory groups had nominally higher risks of all-cause mortality, but these associations were no longer statistically significant after adjustment for eGFR and natural log-transformed UPCR. Only the high-slowly rising proteinuria trajectory group remained statistically significantly associated with all-cause mortality compared with the low-slowly rising proteinuria trajectory group (HR, 1.24; 95% confidence interval, 1.02 to 1.51) in the finally adjusted model. The results were qualitatively unchanged in a sensitivity analysis that adjusted for eGFR and UPCR at baseline (year 0) and for eGFR and UPCR slopes from baseline (year 0) to year 3 (Supplemental Table 5). In an additional sensitivity analysis that re-created the proteinuria trajectory groups from baseline (year 0) to the year 4 visit (Supplemental Figure 2), the associations of proteinuria trajectory groups with future risk of all-cause mortality were qualitatively similar, but the rapidly rising proteinuria group was the only one to reach statistical significance (Supplemental Table 6). The association between the proteinuria trajectory groups and subsequent ESKD were qualitatively unchanged when accounting for the competing risk of death (Supplemental Table 7). Similarly, analyzing the data as a complete case analysis yielded similar results to the primary analysis that incorporated multiple imputation (Supplemental Tables 8 and 9).

**Table 3:** Association of proteinuria trajectory groups with risks of all-cause mortality

Trajectory Groups	No. of Events	Events per 1000 Person-Years	Model 1^a^		Model 2^b^		Model 3^c^	
			Hazard Ratio (95% CI)	*P* Value	Hazard Ratio (95% CI)	*P* Value	Hazard Ratio (95% CI)	*P* Value
Low-slowly rising	397	27.3	Reference	—	Reference	—	Reference	—
High-slowly rising	509	47.9	1.59 (1.38 to 1.84)	<0.001	1.51 (1.30 to 1.75)	<0.001	1.24 (1.02 to 1.51)	0.03
Regressing	36	39.6	1.48 (1.04 to 2.10)	0.03	1.39 (0.98 to 1.99)	0.07	1.33 (0.93 to 1.91)	0.11
Rapidly rising	99	65.2	1.80 (1.43 to 2.28)	<0.001	1.71 (1.25 to 2.17)	<0.001	1.30 (0.97 to 1.75)	0.08

CI, confidence interval.

a Model 1: stratified by clinical centers and adjusted for age, sex, race and ethnicity, household income, education, systolic BP, diabetes, history of cardiovascular disease, body mass index, current smoking, hemoglobin, angiotensin-converting enzyme inhibitors or angiotensin II receptor blockers, *β*-blockers, diuretics, lipid-lowering agents, and antiplatelet agents.

b Model 2: model 1 further adjusted for eGFR.

c Model 3: model 2 further adjusted for natural log-transformed urine protein–creatinine ratio.

## Discussion

In a diverse cohort of over 3000 participants with CKD stages 2–4, latent class trajectory modeling identified four distinct trajectories of proteinuria over 3 years. Most of the study population belonged to two groups characterized by slowly rising levels of proteinuria, one with low levels and the other with high levels. Our analyses also uncovered rapidly rising and regressing proteinuria trajectory groups. Compared with the low-slowly rising proteinuria trajectory group, participants who belonged to the other proteinuria trajectory groups had lower socioeconomic status, a greater prevalence of comorbid conditions, and lower kidney function. All trajectories had significantly higher risks of subsequent ESKD compared with the low-slowly rising proteinuria trajectory group, including those who belonged to the regressing proteinuria trajectory group despite robust reductions in proteinuria over time. By contrast, only the high-slowly rising group exhibited a significantly increased risk of death. The observed risks of ESKD and death were independent of known risk factors, including single-time point eGFR and UPCR values. These findings suggest that subphenotypes of patients exist within a CKD population on the basis of their evolution of proteinuria over time, and these groups have variable risks of ESKD and death.

Spot UPCR measurements facilitate the ability to quantify proteinuria in the clinic and are often measured multiple times during the clinical course of an individual patient.^22^ Levels of proteinuria may change over time because of several factors related to variability, medications that alter proteinuria, changes in kidney function, or natural progression of the underlying disease.^23–26^ Whether these heterogeneous patterns that describe the evolution of proteinuria over time identify subgroups of patients is poorly understood.^7,8^ Our analytic approach leveraged group-based trajectory modeling,^15^ which revealed four distinct proteinuria trajectory groups. Using this approach, a recent publication identified various albuminuria trajectories associated with adverse cardiac mechanics, as assessed by echocardiography in young adults at risk of CVD.^27^ In our study, 90% of the population belonged to groups with slowly rising proteinuria groups (low or high), but two smaller subgroups emerged: rapidly rising and regressing. Participants who belonged to the high-slowly rising and rapidly rising trajectory groups had lower socioeconomic status, greater prevalence of diabetes and CVD, worse systolic BP, greater use of ACEi/ARB and diuretic therapy, and lower eGFR compared with the low-slowly rising proteinuria trajectory group. While participants in the regressing proteinuria trajectory group had a similar socioeconomic and comorbidity risk profile to the high-slowly rising and rapidly rising groups, these participants were younger and had higher rates of ACEi/ARB and diuretic use, lower systolic BP, and higher eGFR. Higher rates of ACEi/ARB use in the high-slowly rising and regressing proteinuria trajectory groups likely reflect recognition by the treating provider of the need to try and lower proteinuria, but it is unclear whether intensification of ACEi/ARB use led to the significant proteinuria reduction seen in the regressing proteinuria trajectory group. While the rapidly rising proteinuria trajectory had a decrease in ACEi/ARB use from baseline to year 3, this seems insufficient to explain the large increase in UPCR. Additional studies are needed to explore factors to explain why the regressing and rapidly rising proteinuria trajectory groups experience dramatic decreases and increases in proteinuria, respectively. Collectively, our results suggest that proteinuria trajectories identify subgroups of patients within a CKD population with varying risk factor profiles.

Single-time point quantification of proteinuria is a well-established risk factor of adverse clinical outcomes.^2,4,28,29^ Despite its measurement multiple times in the clinical course of an individual patient, few studies evaluated the prognostic value of repeated measures of proteinuria.^6–8,26,27^ A prior analysis in the CRIC Study demonstrated that repeated measures of UPCR, including increasing levels over time, were associated with adverse cardiovascular events.^6^ Our analysis showed that each proteinuria trajectory group had increased risks of progression to ESKD compared with the low-slowly rising group independent of multiple risk factors, including eGFR and proteinuria. In addition, the high-slowly rising proteinuria trajectory was at increased risk of death compared with the low-slowly rising group. While it is not surprising that patients with high or rapidly rising levels of proteinuria would be at heightened risk of adverse clinical outcomes, it is interesting that the regressing proteinuria trajectory group was also at increased risk of subsequent ESKD. These findings conflict with a recent meta-analysis of multiple observational studies that demonstrated a 30% decrease in proteinuria after a baseline period of 1–3 years was associated with a decreased risk of future ESKD.^7^ One potential reason for the conflicting findings is that the meta-analysis evaluated change in proteinuria at the population level, whereas our analysis identified a subgroup of patients on the basis of their evolution of proteinuria. As a result, our analytic approach identified a small yet potentially under-recognized subgroup within a population of patients with CKD. While the underlying pathophysiologic rationale for the association between the regressing proteinuria trajectory group and an increased risk of ESKD is not clear, the finding does not seem to be due to significant GFR loss leading to decreased excretion of protein because it remained robust even after adjustment for eGFR at multiple time points. We speculate that patients in the regressing trajectory group have a more complex pathophysiology after reaching a threshold of significant damage to either the podocyte or glomerular basement membrane, which standard clinical biomarkers cannot capture.^24,30^ Owing to the limited regenerative abilities of podocytes and glomerular basement membranes, the seemingly improved level of proteinuria may be a sign of impending nephron loss and subsequent CKD progression.^31^ If our findings are confirmed, additional research is warranted to identify the pathophysiologic process underlying the regressing proteinuria trajectory phenotype.

Strengths of this study include the use of a large, well-characterized CKD cohort with extensive follow-up, statistical methods to identify proteinuria subphenotypes, and rigorous outcome validation through physician adjudication and external databases. This study also has several limitations. Because our study design required participants to have at least two UPCR measurements and survive 3 years without developing ESKD to generate proteinuria trajectory groups in the ascertainment period, our study population differed from the entire CRIC Study population. Although our posterior probabilities of membership in the regressing and rapidly rising proteinuria trajectory groups were ≥0.7, these values were lower than the other proteinuria trajectory groups, which suggests some uncertainty about class membership that may be due to the relatively small number of patients each of these proteinuria trajectory groups. Although our results were similar when using 3 or 4 years to derive proteinuria trajectory groups, this amount of data may not always be available to a treating provider. We were unable to evaluate whether abrupt changes in kidney function due to AKI led to changes in proteinuria, which may alter a participant's membership to a proteinuria trajectory group. In addition, we lack data on the duration of ACEi/ARB use, which can affect levels of proteinuria. In addition, we did not validate our findings in another cohort of patients with CKD, which may limit the generalizability of our results. Because this is an observational study, we cannot exclude the possibility of residual confounding. Further studies involving different cohorts would be beneficial to confirm these findings and assess their applicability across diverse clinical settings.

In conclusion, our study demonstrates that subphenotypes of patients with CKD exist within the population on the basis of their evolution of proteinuria over time. Although most of the participants belonged to trajectories with slowly rising proteinuria levels (low or high), our analysis also uncovered rapidly rising and regressing proteinuria trajectories, each with distinct clinical characteristics. Each proteinuria trajectory group had variable risks of ESKD and death compared with the low-slowly rising proteinuria trajectory group. Future studies are needed to determine whether current or future novel therapies reduce the risk of adverse clinical outcomes in these subphenotypes of patients with CKD on the basis of their prior evolution of proteinuria.

## Supplementary Material

## Data Availability

All de-identified patient-level data are included in the manuscript and/or Supplemental Material.

## Supplemental Material

Supplemental Methods

Supplemental Table 1. Model selection tools.

Supplemental Table 2. Percent missingness of variables.

Supplemental Table 3. Association of proteinuria trajectory groups with risks of ESKD with further adjustment for baseline eGFR, UPCR, and ACEi/ARB use.

Supplemental Table 4. Association of proteinuria trajectory groups with risks of ESKD (using ascertainment period from baseline to year 4 visit).

Supplemental Table 5. Association of proteinuria trajectory groups with risks of all-cause mortality with further adjustment for baseline eGFR, UPCR, and ACEi/ARB use.

Supplemental Table 6. Association of proteinuria trajectory groups with risks of all-cause mortality (using ascertainment period from baseline to year 4 visit).

Supplemental Table 7. Association of proteinuria trajectory groups with risks of ESKD treating death as a competing risk.

Supplemental Table 8. Association of proteinuria trajectory groups with risks of ESKD using a complete case analysis.

Supplemental Table 9. Association of proteinuria trajectory groups with risks of all-cause mortality using a complete case analysis.

Supplemental Figure 1. Distribution of posterior probabilities for proteinuria trajectory group membership.

Supplemental Figure 2. Spaghetti plot illustrating the mean trajectories of UPCR for four distinct subphenotypes from baseline to year 4 visit.
