# Supplementary material for: Associations of Proteinuria Trajectories with Kidney Failure and Death in Individuals with CKD
Source: Kidney360. 2025 Jun 26;6(11):1890–8. doi: 10.34067/KID.0000000849 (PMC12626656; doi:10.34067/KID.0000000849)
Supplement: Supplementary file 1 [file kidney360-6-1890-s001.pdf]

ASN American Society of Nephrology
JASN Journal of the American Society of Nephrology
KIDNEY360 Accessing Our World From Every Angle
CJASN Clinical Journal of the American Society of Nephrology

ASN Journal Disclosure Form

As per ASN journal policy, I have disclosed any financial relationships or commitments I have held in the past 36 months as included below. I have listed my Current Employer below to indicate there is a relationship requiring disclosure. If no relationship exists, my Current Employer is not listed.

A. Aronov reports the following:
Employer: Vanderbilt University Medical Center

I understand that the information above will be published within the journal article, if accepted, and that failure to comply and/or to accurately and completely report the potential financial conflicts of interest could lead to the following: 1) Prior to publication, article rejection, or 2) Post-publication, sanctions ranging from, but not limited to, issuing a correction, reporting the inaccurate information to the authors' institution, banning authors from submitting work to ASN journals for varying lengths of time, and/or retraction of the published work.

Name: Avi Aronov
Manuscript ID: K360-2024-001076R1
Manuscript Title: Associations of Proteinuria Trajectories with Kidney Failure and Death in Individuals with Chronic Kidney Disease
Date of Completion: June 3, 2025
Disclosure Updated Date: May 14, 2025

ASN American Society of Nephrology | JASN Journal of the American Society of Nephrology | KIDNEY360 Accessing Our World From Every Angle | CJASN Clinical Journal of the American Society of Nephrology

ASN Journal Disclosure Form

As per ASN journal policy, I have disclosed any financial relationships or commitments I have held in the past 36 months as included below. I have listed my Current Employer below to indicate there is a relationship requiring disclosure. If no relationship exists, my Current Employer is not listed.

T. Kelly reports the following:
Employer: University of Illinois at Chicago

I understand that the information above will be published within the journal article, if accepted, and that failure to comply and/or to accurately and completely report the potential financial conflicts of interest could lead to the following: 1) Prior to publication, article rejection, or 2) Post-publication, sanctions ranging from, but not limited to, issuing a correction, reporting the inaccurate information to the authors' institution, banning authors from submitting work to ASN journals for varying lengths of time, and/or retraction of the published work.

Name: Tanika Kelly
Manuscript ID: K360-2024-001076R1
Manuscript Title: Associations of Proteinuria Trajectories with Kidney Failure and Death in Individuals with Chronic Kidney Disease
Date of Completion: April 11, 2025
Disclosure Updated Date: April 11, 2025

ASN
American Society of Nephrology
JASN
Journal of the American Society of Nephrology
KIDNEY360
Accessing Our World From Every Angle
CJASN
Clinical Journal of the American Society of Nephrology

ASN Journal Disclosure Form

As per ASN journal policy, I have disclosed any financial relationships or commitments I have held in the past 36 months as included below. I have listed my Current Employer below to indicate there is a relationship requiring disclosure. If no relationship exists, my Current Employer is not listed.

J. Lash reports the following:
Employer: University of Illinois at Chicago

I understand that the information above will be published within the journal article, if accepted, and that failure to comply and/or to accurately and completely report the potential financial conflicts of interest could lead to the following: 1) Prior to publication, article rejection, or 2) Post-publication, sanctions ranging from, but not limited to, issuing a correction, reporting the inaccurate information to the authors' institution, banning authors from submitting work to ASN journals for varying lengths of time, and/or retraction of the published work.

Name: James P. Lash
Manuscript ID: K360-2024-001076R1
Manuscript Title: Associations of Proteinuria Trajectories with Kidney Failure and Death in Individuals with Chronic Kidney Disease
Date of Completion: April 10, 2025
Disclosure Updated Date: February 20, 2025

ASN
American Society of Nephrology
JASN
Journal of the American Society of Nephrology
KIDNEY360
Accessing Our World From Every Angle
CJASN
Clinical Journal of the American Society of Nephrology

ASN Journal Disclosure Form

As per ASN journal policy, I have disclosed any financial relationships or commitments I have held in the past 36 months as included below. I have listed my Current Employer below to indicate there is a relationship requiring disclosure. If no relationship exists, my Current Employer is not listed.

A. Ricardo has nothing to disclose.

I understand that the information above will be published within the journal article, if accepted, and that failure to comply and/or to accurately and completely report the potential financial conflicts of interest could lead to the following: 1) Prior to publication, article rejection, or 2) Post-publication, sanctions ranging from, but not limited to, issuing a correction, reporting the inaccurate information to the authors' institution, banning authors from submitting work to ASN journals for varying lengths of time, and/or retraction of the published work.

Name: Ana C. Ricardo
Manuscript ID: K360-2024-001076R1
Manuscript Title: Associations of Proteinuria Trajectories with Kidney Failure and Death in Individuals with Chronic Kidney Disease
Date of Completion: April 11, 2025
Disclosure Updated Date: November 11, 2024

ASN
American Society of Nephrology
JASN
Journal of the American Society of Nephrology
KIDNEY360
Accessing Our World From Every Angle
CJASN
Clinical Journal of the American Society of Nephrology

ASN Journal Disclosure Form

As per ASN journal policy, I have disclosed any financial relationships or commitments I have held in the past 36 months as included below. I have listed my Current Employer below to indicate there is a relationship requiring disclosure. If no relationship exists, my Current Employer is not listed.

A. Srivastava reports the following:
Employer: University of Illinois Chicago; Consultancy: CVS Caremark; Novo Nordisk; and Honoraria: Horizon Therapeutics PLC/Amgen; AstraZeneca; Bayer; FNIH; University of Chicago; University of Washington; Endeavor Health; American Diabetes Association.

I understand that the information above will be published within the journal article, if accepted, and that failure to comply and/or to accurately and completely report the potential financial conflicts of interest could lead to the following: 1) Prior to publication, article rejection, or 2) Post-publication, sanctions ranging from, but not limited to, issuing a correction, reporting the inaccurate information to the authors' institution, banning authors from submitting work to ASN journals for varying lengths of time, and/or retraction of the published work.

Name: Anand Srivastava
Manuscript ID: K360-2024-001076R1
Manuscript Title: Associations of Proteinuria Trajectories with Kidney Failure and Death in Individuals with Chronic Kidney Disease
Date of Completion: April 11, 2025
Disclosure Updated Date: April 11, 2025

ASN
American Society of Nephrology
JASN
Journal of the American Society of Nephrology
KIDNEY360
Accessing Our World From Every Angle
CJASN
Clinical Journal of the American Society of Nephrology

ASN Journal Disclosure Form

As per ASN journal policy, I have disclosed any financial relationships or commitments I have held in the past 36 months as included below. I have listed my Current Employer below to indicate there is a relationship requiring disclosure. If no relationship exists, my Current Employer is not listed.

A. Verma reports the following:
Employer: Boston University School of Medicine/Boston Medical Center; Research Funding: American Heart Association (Dallas, US); GRANT_NUMBER: 24CDA1274501; and Advisory or Leadership Role: Editorial Board: Therapeutic Advances in Endocrinology and Metabolism, BMC Medicine, BMC Nephrology, and Therapeutic Advances in Chronic Disease.

I understand that the information above will be published within the journal article, if accepted, and that failure to comply and/or to accurately and completely report the potential financial conflicts of interest could lead to the following: 1) Prior to publication, article rejection, or 2) Post-publication, sanctions ranging from, but not limited to, issuing a correction, reporting the inaccurate information to the authors' institution, banning authors from submitting work to ASN journals for varying lengths of time, and/or retraction of the published work.

Name: Ashish Verma
Manuscript ID: K360-2024-001076R1
Manuscript Title: "Associations of Proteinuria Trajectories with Kidney Failure and Death in Individuals with Chronic Kidney Disease,"
Date of Completion: May 2, 2025
Disclosure Updated Date: November 11, 2024

ASN American Society of Nephrology
JASN Journal of the American Society of Nephrology
KIDNEY360 Accessing Our World From Every Angle
CJASN Clinical Journal of the American Society of Nephrology

ASN Journal Disclosure Form

As per ASN journal policy, I have disclosed any financial relationships or commitments I have held in the past 36 months as included below. I have listed my Current Employer below to indicate there is a relationship requiring disclosure. If no relationship exists, my Current Employer is not listed.

S. Waikar reports the following:
Employer: Google (spouse); Consultancy: Wolters Kluewer, Aditum, Bain, CANbridge, Delix, Ikena, Merck, Mineralys, Motric Bio, Ono Pharma, PepGen, Strataca, Vertex; Research Funding: Vertex, Pfizer, JNJ, Natera; and Other Interests or Relationships: expert witness for litigation related to dialysis lab testing (Davita), PPIs (Pfizer), PFAO exposure (Dechert).

I understand that the information above will be published within the journal article, if accepted, and that failure to comply and/or to accurately and completely report the potential financial conflicts of interest could lead to the following: 1) Prior to publication, article rejection, or 2) Post-publication, sanctions ranging from, but not limited to, issuing a correction, reporting the inaccurate information to the authors' institution, banning authors from submitting work to ASN journals for varying lengths of time, and/or retraction of the published work.

Name: Sushrut S. Waikar
Manuscript ID: K360-2024-001076R1
Manuscript Title: Associations of Proteinuria Trajectories with Kidney Failure and Death in Individuals with Chronic Kidney Disease
Date of Completion: May 8, 2025
Disclosure Updated Date: March 24, 2025
