# Supplementary material for: Associations of Proteinuria Trajectories with Kidney Failure and Death in Individuals with CKD
Source: Kidney360. 2025 Jun 26;6(11):1890–8. doi: 10.34067/KID.0000000849 (PMC12626656; doi:10.34067/KID.0000000849)

Supplemental Materials

Associations of Proteinuria Trajectories with Kidney Failure and Death in Individuals with Chronic Kidney Disease

Avi G. Aronov MD[1], Ashish Verma MD[2], Ana C. Ricardo MD[3], Tanika N. Kelly PhD MPH[3], Sushrut S. Waikar MD[2], James P. Lash MD[3], Anand Srivastava MD MPH[3], and the CRIC Study Investigators[†]

1. Department of Medicine, University of Illinois Chicago, Chicago, IL 60612, USA.
2. Section of Nephrology, Department of Medicine, Boston Medical Center and Boston University Chobanian & Avedisian School of Medicine, Boston, Massachusetts 02118, USA.
3. Division of Nephrology, Department of Medicine, University of Illinois Chicago, Chicago, IL 60612, USA.

[†]CRIC Study Investigators: Amanda H. Anderson PhD MPH, Lawrence J. Appel MD MPH, Jing Chen MD MMSc MSc, Debbie L. Cohen MD, Laura M. Dember MD, Alan S. Go MD, Robert G. Nelson MD PhD MS, Mahboob Rahman MD, Panduranga S. Rao MD, Vallabh O. Shah PhD MS, Mark L. Unruh MD MS.

Supplemental Tables: 9

Supplemental Figures: 2

Corresponding author:

Anand Srivastava, MD, MPH
Email: asrivast@uic.edu

Supplemental Methods

To account for missing covariate data, we used the 'mice package' for multiple imputations using predictive mean matching. We generated 10 imputed datasets and 100 interations with the assumptions that the data was missing at random. We combined the test results across the imputed datasets using the rules of Rubin.[1]

1. Rubin, D: *Multiple Imputation for Nonresponse in Surveys,* New York, John Wiley and Sons, 2004.

Supplemental Table 1. Model selection tools

No. of groups	Average Posterior Probability Assignment[a]	Odds of Correct Classification[b]	Relative Entropy[c]	BIC[d]
**2**	0.944, 0.855	13.5, 7.36	0.647	32,467
**3**	0.858, 0.887, 0.795	7.92, 8.73, 37.6	0.688	32,105
**4**	0.858, 0.874, 0.819, 0.780	7.89, 8.58, 104, 43.3	0.734	31,879
**5**	0.815, 0.856, 0.821, 0.826, 0.719	65.6, 15.2, 98.8, 9.62, 6.57	0.694	31,813
**6**	0.731, 0.822, 0.829, 0.848, 0.719, 0.760	6.69, 9.43, 100, 15.7, 137, 54.1	0.716	31,752

[a]Considered to have a good fit when all values ≥0.7.
[b]Considered to have a good fit when all values ≥5.
[c]Models closer to 1 considered to have a better fit.
[d]Smaller values indicate a better model.

Supplemental Table 2. Percent missingness of variables

Characteristics	Percent Missingness (%)
**Age, years**	–
**Male**	–
**Race/Ethnicity**	–
**Household income, dollars**	–
**Education level**	0.1
**Current smoking**	–
**Body mass index, kg/m^2**	3.6
**Systolic blood pressure, mmHg**	0.4
**Diabetes mellitus**	–
**CVD[a]**	–
**ACEi/ARB**	0.4
**Diuretic**	0.4
**Lipid-lowering medication**	0.4
**Antiplatelet**	0.4
**Beta-blocker**	0.4
**Hemoglobin, g/L**	2.2
**eGFR, ml/min/1.73m^2 (baseline)**	–
**eGFR, ml/min/1.73m^2 (year 3)**	0.8
**UPCR, g/g (baseline)**	–
**UPCR, g/g (year 3)**	–

ACEi=angiotensin-converting enzyme inhibitor, ARB=angiotensin II receptor blocker, CVD=cardiovascular disease, eGFR=estimated glomerular filtration rate.

All variables ascertained at the year 3 visit unless noted otherwise.

[a]Defined as either congestive heart failure, myocardial infarction, cerebrovascular accident, or peripheral vascular disease.

Supplemental Table 3. Association of proteinuria trajectory groups with risks of ESKD with further adjustment for baseline eGFR, UPCR, and ACEi/ARB use

	Low-Slowly Rising		High-Slowly Rising		Regressing		Rapidly Rising	
	Hazard Ratio (95% Confidence Interval)	P Value	Hazard Ratio (95% Confidence Interval)	P Value	Hazard Ratio (95% Confidence Interval)	P Value	Hazard Ratio (95% Confidence Interval)	P Value
Model 1	Reference	—	8.07 (6.30 – 10.3)	<0.001	5.35 (3.54 – 8.06)	<0.001	9.59 (6.97 – 13.2)	<0.001
Model 2	Reference	—	4.59 (3.56 – 5.90)	<0.001	2.67 (1.77 – 4.04)	<0.001	6.77 (4.89 – 9.38)	<0.001
Model 3	Reference	—	1.55 (1.13 – 2.11)	<0.001	2.23 (1.46 – 3.40)	<0.001	1.54 (1.03 – 2.31)	0.04
Model 4	Reference	—	1.42 (1.03 – 2.97)	0.03	1.75 (1.06 – 2.91)	0.03	1.71 (1.12 – 2.62)	0.01
Model 5	Reference	—	1.45 (1.04 – 2.00)	0.03	1.80 (1.08 – 3.00)	0.02	1.72 (1.12 – 2.62)	0.01

[a]Model 1: stratified by clinical centers and adjusted for age, sex, race/ethnicity, household income, education, systolic blood pressure, diabetes, history of cardiovascular disease, body mass index, current smoking, hemoglobin, angiotensin-converting enzyme inhibitor or angiotensin II receptor blockers, beta-blockers, diuretics, lipid-lowering agents, and antiplatelet agents.

[b]Model 2: Model 1 further adjusted for year 3 eGFR.

[c]Model 3: Model 2 further adjusted for year 3 natural log-transformed UPCR.

[d]Model 4: Model 3 further adjusted for baseline visit (year 0) eGFR and natural log-transformed UPCR.

[e]Model 5: Model 4 further adjusted for baseline visit (year 0) angiotensin-converting enzyme inhibitor or angiotensin II receptor blockers.

Supplemental Table 4. Association of proteinuria trajectory groups with risks of ESKD (using ascertainment period from baseline to year 4 visit)

Trajectory Groups	No. of events	Events per 1000 person-years	Model 1[a]		Model 2[b]		Model 3[c]	
			Hazard Ratio (95% Confidence Interval)	P Value	Hazard Ratio (95% Confidence Interval)	P Value	Hazard Ratio (95% Confidence Interval)	P Value
Low-Slowly Rising	65	6.08	Reference	—	Reference	—	Reference	—
High-Slowly Rising	397	66.2	8.06 (5.96 – 10.9)	<0.001	4.69 (3.45 – 6.38)	<0.001	1.49 (1.01 – 2.19)	0.04
Regressing	39	46.0	5.17 (3.32 – 8.05)	<0.001	2.62 (1.67 – 4.12)	<0.001	1.69 (1.05 – 2.70)	0.03
Rapidly Rising	71	86.3	10.2 (6.79 – 15.4)	<0.001	6.89 (4.55 – 10.4)	<0.001	1.91 (1.17 – 3.11)	0.01

[a]Model 1: stratified by clinical centers and adjusted for age, sex, race/ethnicity, household income, education, systolic blood pressure, diabetes, history of cardiovascular disease, body mass index, current smoking, hemoglobin, angiotensin-converting enzyme inhibitor or angiotensin II receptor blockers, beta-blockers, diuretics, lipid-lowering agents, and antiplatelet agents.

[b]Model 2: Model 1 further adjusted for eGFR.

[c]Model 3: Model 2 further adjusted for natural log-transformed UPCR.

Supplemental Table 5. Association of proteinuria trajectory groups with risks of all-cause mortality with further adjustment for baseline eGFR, UPCR, and ACEi/ ARB use

	Low Slowly Rising		High-Slowly Rising		Regressing		Rapidly Rising	
	Hazard Ratio (95% Confidence Interval)	P Value	Hazard Ratio (95% Confidence Interval)	P Value	Hazard Ratio (95% Confidence Interval)	P Value	Hazard Ratio (95% Confidence Interval)	P Value
Model 1	Reference	—	1.59 (1.38 – 1.84)	<0.001	1.48 (1.04 – 2.10)	0.029	1.80 (1.43 – 2.28)	<0.001
Model 2	Reference	—	1.51 (1.30 – 1.75)	<0.001	1.39 (0.98 – 1.99)	0.066	1.71 (1.25 – 2.17)	<0.001
Model 3	Reference	—	1.24 (1.02 – 1.51)	0.030	1.33 (0.93 – 1.91)	0.11	1.30 (0.97 – 1.75)	0.083
Model 4	Reference	—	1.29 (1.04 – 1.58)	0.018	1.55 (1.01 – 2.38)	0.043	1.21 (0.88 – 1.64)	0.24
Model 5	Reference	—	1.29 (1.05 – 1.60)	0.015	1.50 (0.98 – 2.30)	0.065	1.26 (0.92 – 1.73)	0.15

[a]Model 1: stratified by clinical centers and adjusted for age, sex, race/ethnicity, household income, education, systolic blood pressure, diabetes, history of cardiovascular disease, body mass index, current smoking, hemoglobin, angiotensin-converting enzyme inhibitor or angiotensin II receptor blockers, beta-blockers, diuretics, lipid-lowering agents, and antiplatelet agents.

[b]Model 2: Model 1 further adjusted for year 3 eGFR.

[c]Model 3: Model 2 further adjusted for year 3 natural log-transformed UPCR.

[d]Model 4: Model 3 further adjusted for baseline visit (year 0) eGFR and natural log-transformed UPCR.

[e]Model 5: Model 4 further adjusted for baseline visit (year 0) angiotensin-converting enzyme inhibitor or angiotensin II receptor blockers (year 0).

Supplemental Table 6. Association of proteinuria trajectory groups with risks of all-cause mortality (using ascertainment period from baseline to year 4 visit)

Trajectory Groups	No. of Events	Events per 1000 Person-Years	Model 1[a]		Model 2[b]		Model 3[c]	
			Hazard Ratio (95% Confidence Interval)	P Value	Hazard Ratio (95% Confidence Interval)	P Value	Hazard Ratio (95% Confidence Interval)	P Value
Low-Slowly Rising	267	24.8	Reference	—	Reference	—	Reference	—
High-Slowly Rising	298	39.6	1.56 (1.30 – 1.86)	<0.001	1.44 (1.20 – 1.74)	<0.001	1.22 (0.95 – 1.57)	0.12
Regressing	37	37.8	1.51 (1.06 – 2.17)	0.022	1.39 (0.97 – 2.00)	0.073	1.32 (0.92 – 1.91)	0.13
Rapidly Rising	67	65.9	2.21 (1.65 – 2.95)	<0.001	2.05 (1.53 – 2.75)	<0.001	1.68 (1.18 – 2.40)	0.004

[a]Model 1: stratified by clinical centers and adjusted for age, sex, race/ethnicity, household income, education, systolic blood pressure, diabetes, history of cardiovascular disease, body mass index, current smoking, hemoglobin, angiotensin-converting enzyme inhibitor or angiotensin II receptor blockers, beta-blockers, diuretics, lipid-lowering agents, and antiplatelet agents.
[b]Model 2: Model 1 further adjusted for eGFR.
[c]Model 3: Model 2 further adjusted for natural log-transformed UPCR.

Supplemental Table 7. Association of proteinuria trajectory groups with risks of ESKD treating death as a competing risk

Trajectory Groups	No. of events	Events per 1000 person-years	Model 1[a]		Model 2[b]		Model 3[c]	
			Hazard Ratio (95% Confidence Interval)	P Value	Hazard Ratio (95% Confidence Interval)	P Value	Hazard Ratio (95% Confidence Interval)	P Value
Low-slowly Rising	69	5.60	Reference	—	Reference	—	Reference	—
High-slowly Rising	381	59.8	7.76 (5.60 – 10.6)	<0.001	5.43 (3.87 – 7.60)	<0.001	1.90 (1.22 – 2.96)	0.005
Regressing	29	48.9	4.83 (3.19 – 7.33)	<0.001	2.48 (1.62 – 3.80)	<0.001	2.13 (1.34 – 3.40)	0.001
Rapidly rising	68	85.9	6.72 (5.28 – 8.55)	<0.001	3.68 (2.88 – 4.70)	<0.001	1.64 (1.18 – 2.27)	0.003

[a]Model 1: stratified by clinical centers and adjusted for age, sex, race/ethnicity, household income, education, systolic blood pressure, diabetes, history of cardiovascular disease, body mass index, current smoking, hemoglobin, angiotensin-converting enzyme inhibitor or angiotensin II receptor blockers, beta-blockers, diuretics, lipid-lowering agents, and antiplatelet agents.

[b]Model 2: Model 1 and further adjusted for eGFR.

[c]Model 3: Model 2 and further adjusted for natural log-transformed UPCR.

Supplemental Table 8. Association of proteinuria trajectory groups with risks of ESKD using a complete case analysis

Trajectory Groups	No. of events	Events per 1000 person-years	Model 1[a]		Model 2[b]		Model 3[c]	
			Hazard Ratio (95% Confidence Interval)	P Value	Hazard Ratio (95% Confidence Interval)	P Value	Hazard Ratio (95% Confidence Interval)	P Value
Low-slowly Rising	77	5.58	Reference	—	Reference	—	Reference	—
High-slowly Rising	475	64.1	8.30 (6.46 – 10.7)	<0.001	4.70 (3.63 – 6.07)	<0.001	1.59 (1.16 – 2.19)	0.004
Regressing	32	47.7	5.68 (3.72 – 8.69)	<0.001	2.75 (1.79 – 4.23)	<0.001	2.23 (1.44 – 3.45)	0.003
Rapidly rising	87	95.9	9.83 (7.11 – 13.6)	<0.001	7.05 (5.08 – 9.80)	<0.001	1.63 (1.07 – 2.45)	0.02

[a]Model 1: stratified by clinical centers and adjusted for age, sex, race/ethnicity, household income, education, systolic blood pressure, diabetes, history of cardiovascular disease, body mass index, current smoking, hemoglobin, angiotensin-converting enzyme inhibitor or angiotensin II receptor blockers, beta-blockers, diuretics, lipid-lowering agents, and antiplatelet agents.

[b]Model 2: Model 1 and further adjusted for eGFR.

[c]Model 3: Model 2 and further adjusted for natural log-transformed UPCR.

Supplemental Table 9. Association of proteinuria trajectory groups with risks of all-cause mortality using a complete case analysis

Trajectory Groups	No. of events	Events per 1000 person-years	Model 1[a]		Model 2[b]		Model 3[c]	
			Hazard Ratio (95% Confidence Interval)	P Value	Hazard Ratio (95% Confidence Interval)	P Value	Hazard Ratio (95% Confidence Interval)	P Value
Low-slowly Rising	340	24.6	Reference	—	Reference	—	Reference	—
High-slowly Rising	280	37.8	1.54 (1.30 – 1.83)	<0.001	1.50 (1.26 – 1.79)	<0.001	1.14 (0.90 – 1.45)	0.28
Regressing	18	26.8	1.33 (0.82 – 2.16)	0.25	1.28 (0.79 – 2.08)	0.32	1.19 (0.73 – 1.95)	0.47
Rapidly rising	49	54.0	2.18 (1.59 – 2.98)	<0.001	2.15 (1.57 – 2.94)	<0.001	1.44 (0.97 – 2.14)	0.07

[a]Model 1: stratified by clinical centers and adjusted for age, sex, race/ethnicity, household income, education, systolic blood pressure, diabetes, history of cardiovascular disease, body mass index, current smoking, hemoglobin, angiotensin-converting enzyme inhibitor or angiotensin II receptor blockers, beta-blockers, diuretics, lipid-lowering agents, and antiplatelet agents.

[b]Model 2: Model 1 further adjusted for eGFR.

[c]Model 3: Model 2 further adjusted for natural log-transformed UPCR.

Supplemental Figure 1. Distribution of posterior probabilities for proteinuria trajectory group membership.

The histogram depicts the frequency of participants' highest posterior probability of belonging to one of the four identified proteinuria trajectory sub-phenotypes. The x-axis is segmented into intervals of posterior probability ranges, while the y-axis denotes the percentage of each sub-phenotype belonging to the given interval.

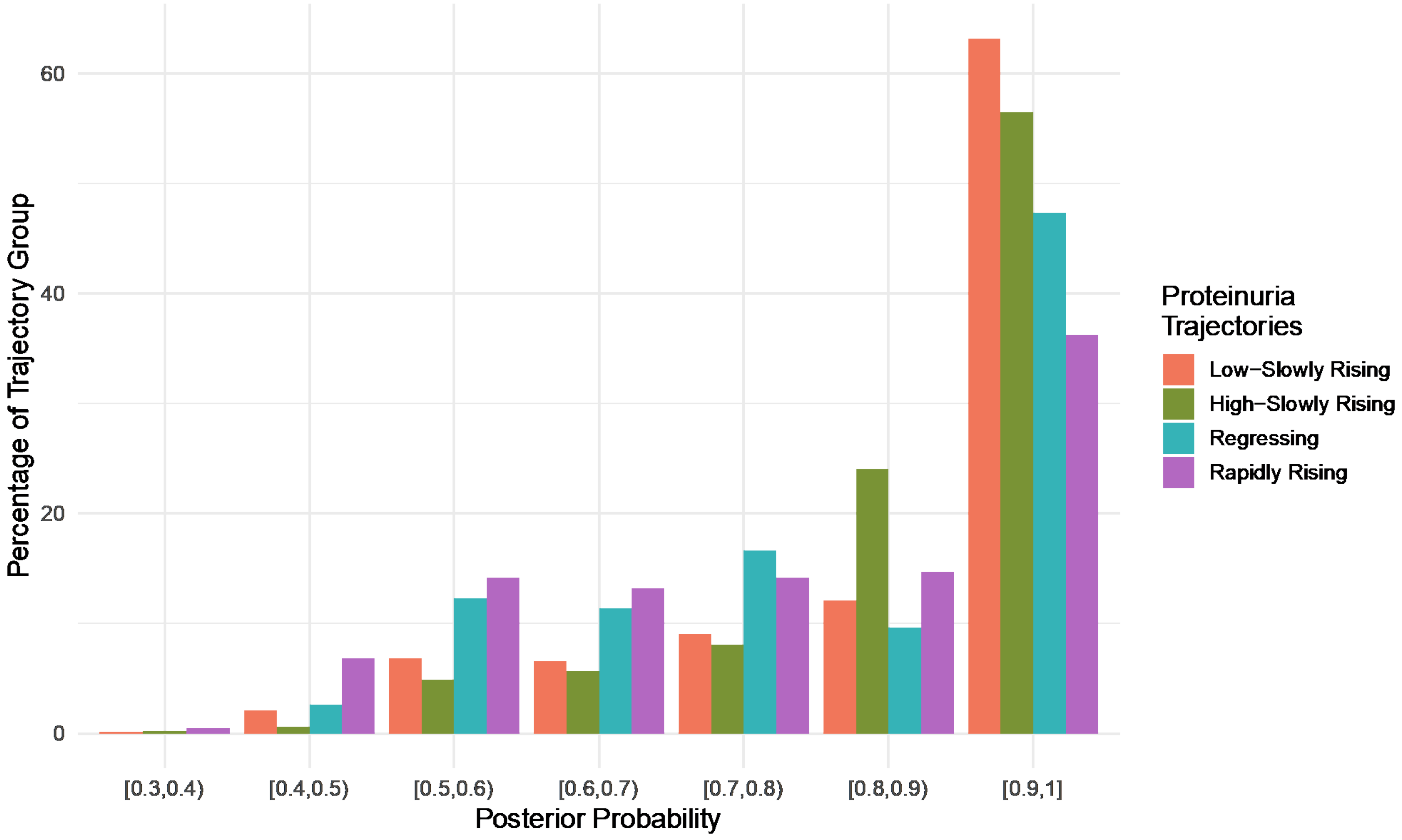

Supplemental Figure 2. Spaghetti plot illustrating the mean trajectories of UPCR for four distinct sub-phenotypes from baseline to year 4 visit.

The analysis included 2,322 participants, categorized into low-slowly rising (n=1,106), high-slowly rising (n=951), regressing (n=118), and rapidly rising (n=147) groups. The solid lines represent the mean natural log-transformed UPCR within each trajectory, while the shaded ribbons indicate the variation within one standard deviation of the mean. The x-axis denotes the follow-up time in years, and the y-axis reflects the log-transformed ratio.

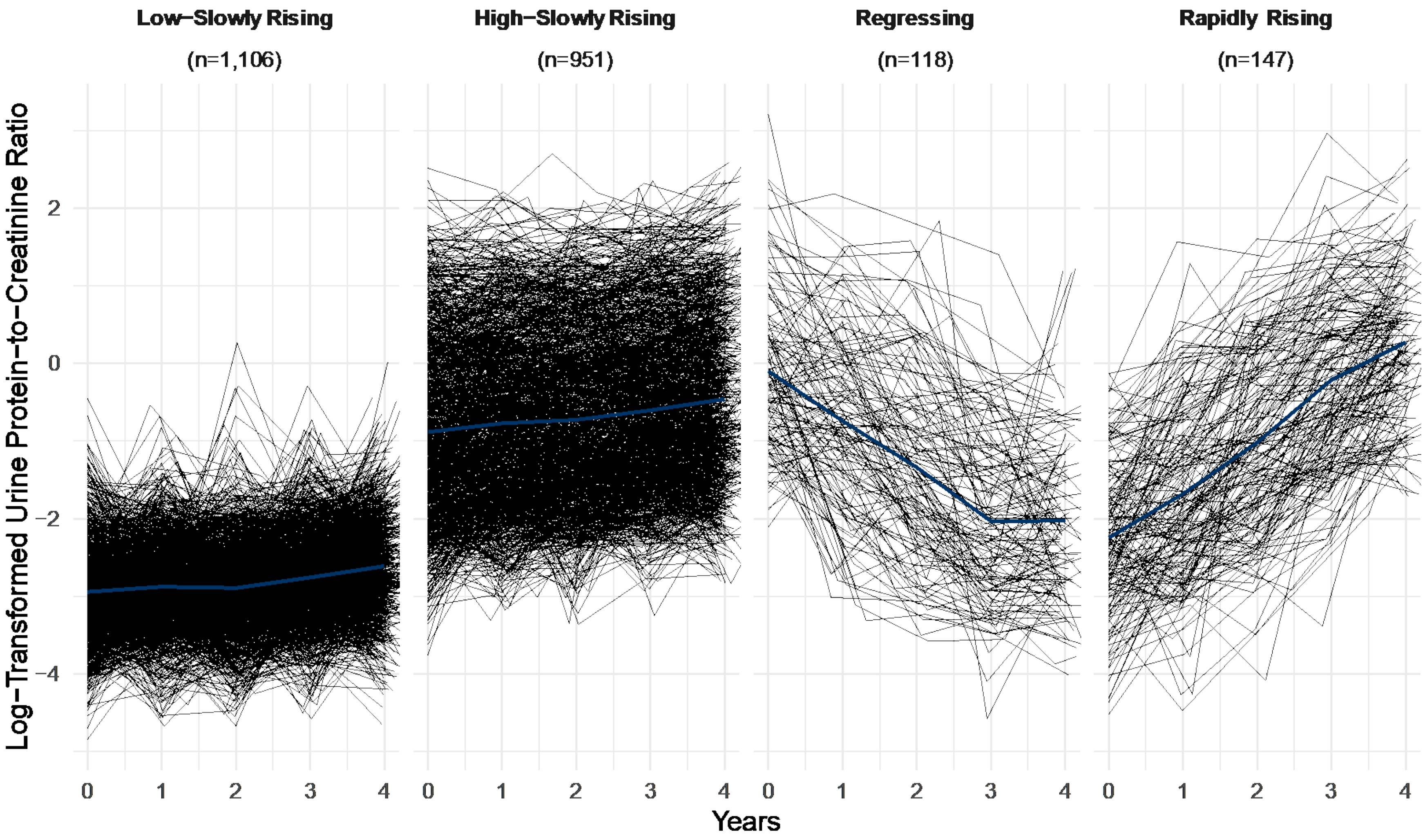

Supplement: Supplementary file 2 [file kidney360-6-1890-s002.pdf]
